# N-glycan mediated adhesion strengthening during pathogen-receptor binding revealed by cell-cell force spectroscopy

**DOI:** 10.1038/s41598-017-07220-w

**Published:** 2017-07-27

**Authors:** Joost te Riet, Ben Joosten, Inge Reinieren-Beeren, Carl G. Figdor, Alessandra Cambi

**Affiliations:** 1 Department of Tumor Immunology, Radboud Institute for Medical Life Sciences, Radboud University Medical Center, Grooteplein Zuid 26-28, 6525 GA Nijmegen, The Netherlands; 2 Department of Cell Biology, Radboud Institute for Molecular Life Sciences, Radboud University Medical Center, Geert Grooteplein Zuid 26-28, 6525 GA Nijmegen, The Netherlands; 3 Present Address: Department of Radiology and Nuclear Medicine, Radboud University Medical Center, Geert Grooteplein Zuid 10, 6525 GA Nijmegen, The Netherlands

## Abstract

Glycan-protein lateral interactions have gained increased attention as important modulators of receptor function, by regulating surface residence time and endocytosis of membrane glycoproteins. The pathogen-recognition receptor DC-SIGN is highly expressed at the membrane of antigen-presenting dendritic cells, where it is organized in nanoclusters and binds to different viruses, bacteria and fungi. We recently demonstrated that DC-SIGN *N*-glycans spatially restrict receptor diffusion within the plasma membrane, favoring its internalization through clathrin-coated pits. Here, we investigated the involvement of the *N*-glycans of DC-SIGN expressing cells on pathogen binding strengthening when interacting with *Candida* fungal cells by using atomic force microscope (AFM)-assisted single cell-pathogen adhesion measurements. The use of DC-SIGN mutants lacking the *N*-glycans as well as blocking glycan-mediated lateral interactions strongly impaired cell stiffening during pathogen binding. Our findings demonstrate for the first time the direct involvement of the cell membrane glycans in strengthening cell-pathogen interactions. This study, therefore, puts forward a possible role for the glycocalyx as extracellular cytoskeleton contributing, possibly in connection with the intracellular actin cytoskeleton, to optimize strengthening of cell-pathogen interactions in the presence of mechanical forces.

## Introduction

Understanding how the plasma membrane composition and its spatial organization control receptor function is an area of intense investigation. Nanoscale clustering, localization within specific membrane lipid domains, anchoring to cortical actin structures and lateral mobility patterns have been shown to modulate the function of membrane receptors including integrins^1–3^, growth factor receptors^4, 5^, immune receptors^6–8^, as well as pathogen-recognition receptors (PRRs)^9–11^. Glycosylation of plasma membrane proteins is known to increase their residence time at the plasma membrane, facilitate cell-cell adhesion through lectin-glycan interactions, and regulate endocytosis of receptors such as EGFR or CD44^12, 13^. If in the latter case, lateral interactions between plasma membrane proteins within the glycan-protein layer of a cell, *i.e*. glycocalyx, also play a role in regulating adhesion is still mainly unknown.

**Figure 1:**
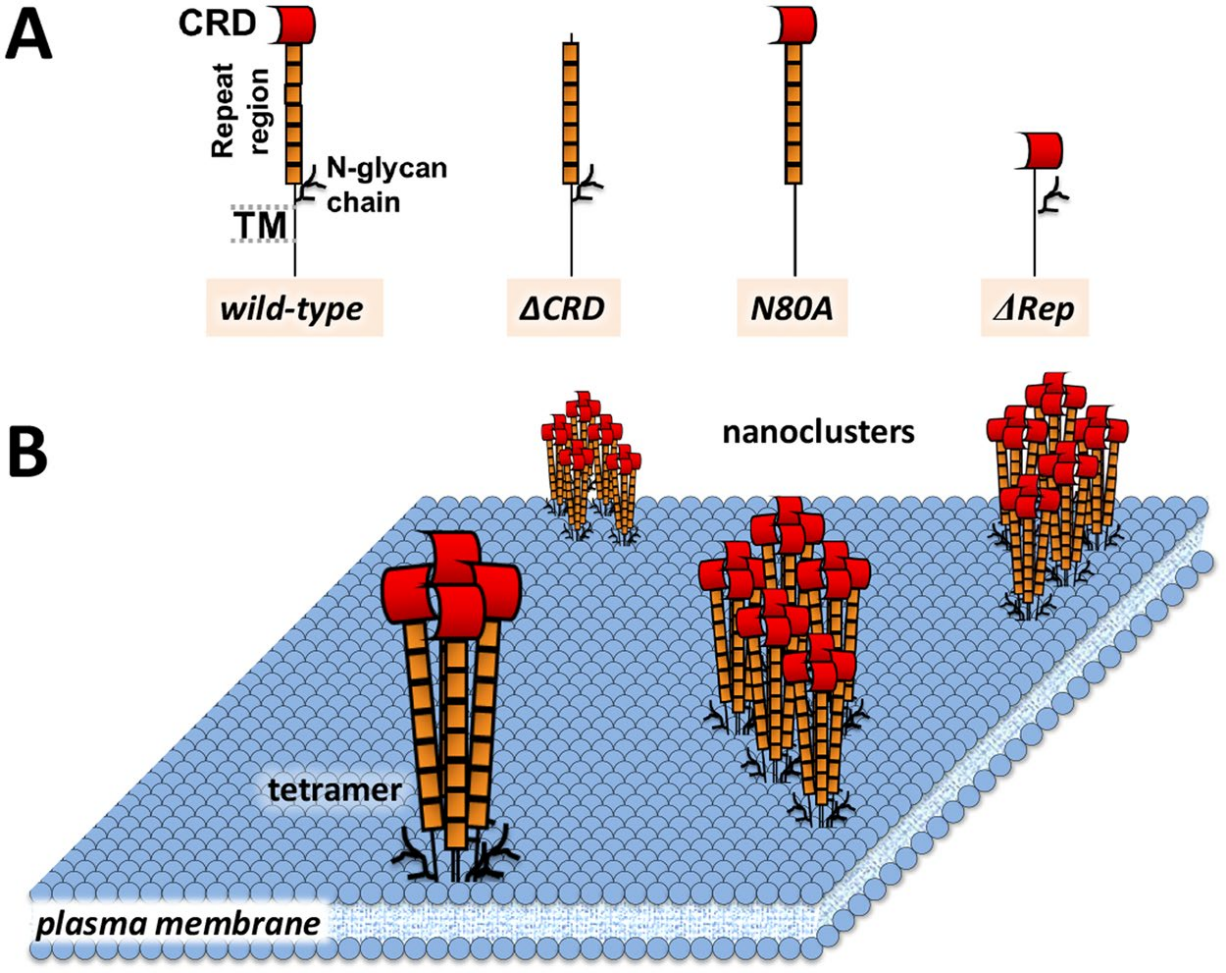
Structure and membrane nanoscale organization of DC-SIGN. (**A**) DC-SIGN is type-II C-type lectin (N-terminus is intracellular) that has an extracellular Carbohydrate Recognition Domain (CRD), a region of 7.5 amino acid repeats, preceded by a site for *N*-glycosylation (amino acid N80), a transmembrane region and a short cytoplasmic tail. In this study, besides the wild-type, several mutants of DC-SIGN were used: DC-SIGN-ΔCRD, which lacks the ligand binding domain; DC-SIGN-N80A, which carries a point mutation that abolish *N*-glycosylation; and DC-SIGN-ΔRep, which only lacks the repeat region^25^. (**B**) Except for DC-SIGN-ΔRep, all the other variants can form tetramers and organize in small nanoclusters within the plasma membrane^10^.

Dendritic Cell-Specific ICAM-3-Grabbing Non-Integrin (DC-SIGN; CD209) is a type II plasma membrane PRR abundantly expressed in antigen-presenting cells such as dendritic cells (DCs) and activated macrophages^14, 15^. As member of the C-type Lectin Receptor (CLR) family, DC-SIGN binds a plethora of pathogens, ranging from viruses like HIV-1^16^, ebola virus^17^, and hepatitis C virus^18^, to larger pathogens like *Mycobacterium tuberculosis* ^19^ and *Candida albicans* ^20^. DC-SIGN directly binds glycan structures exposed at the pathogen surface. Structurally, DC-SIGN is a tetramer, with each monomer having a long extracellular part composed by a carbohydrate recognition domain (CRD), a 7.5 tandem repeat of 23 amino acids forming the neck region, and a transmembrane part followed by a cytoplasmic tail containing a recycling and internalization motif (Fig. 1)^21, 22^. The neck region (*i.e*. repeat region) of DC-SIGN is responsible for its tetramerization^23^ enabling the clustering of CRDs to present multiple binding sites and increasing the interaction strength with specific ligands^24^. In addition, DC-SIGN harbors a glycosylation motif at the membrane proximal part of the repeat region, with the Asn80 residue undergoing *N*-glycosylation^25^. Moreover, DC-SIGN forms nanoclusters at the cell membrane (Fig. 1), which are crucial for virus binding^9, 26, 27^. Similarly, DC-SIGN nanocluster formation and/or stability do not require interactions with the cortical cytoskeleton or association with tetraspanins^28^. We recently showed that impaired *N*-glycosylation of DC-SIGN significantly altered the lateral mobility pattern and ultimately the capacity of the receptor to follow clathrin-mediated endocytosis^29^. Considering the importance of lateral mobility for receptor-ligand interactions, we hypothesize a role of plasma membrane glycans in regulating the adhesion strength between receptors and pathogen.

Adhesive interactions between cells, including immune cells and pathogens, mostly occur in a dynamic environment caused by, for example, flowing body fluids, and thus bonds will experience mechanical loading. Cell adhesion can be regulated by mechanisms such as (*i*) up- or down-regulation of the surface expression of cell adhesion molecules (CAMs), (*ii*) changing affinity of the CAM, or (*iii*) clustering of CAMs^30^. The latter two can be influenced by interactions of the cytoplasmic part of the CAM with the underlying actin cytoskeleton^7, 31^. Since adhesion is not a static process, though dynamic, experimental methods probing adhesive interactions between single cells might provide detailed insight into the complexity of adhesion regulation. A very promising technique to explore this is atomic force microscopy (AFM)-assisted single-cell force spectroscopy (SCFS)^32, 33^ that can probe the interaction between a cell and isolated CAMs^33–36^ or extracellular matrix proteins^37–39^, between cells^40–42^, and between cell and pathogen^43–45^. Moreover, hybrid AFM-light microscopy allows manipulating cells with submicron accuracy while monitoring cellular responses and allows measuring interactions forces up to the single-molecule level. AFM-assisted SCFS with a single *C. albicans* fungal cell on an AFM cantilever allowed us to study the recognition strength of DC-SIGN under mechanical load showing that DC-SIGN specifically discriminates between carbohydrate moieties in the cell wall of the fungus *Candida albicans* that have similar chemical composition but slightly different structures^34^. Infections caused by *Candida* are the main cause of mortality due to invasive mycotic diseases for severely immunocompromised patients^20, 46, 47^. Studies on the initial binding of different *Candida* species (*e.g., C. albicans*, *C. paropsilosis*, and *C. dubliniensis*) with DCs showed that DC-SIGN as well as the macrophage mannose receptor (MMR; CD206) are involved with the detection of fungal *Candida* cells^20, 47^.

In this study, we used cell-cell force spectroscopy (CCFS) to investigate the involvement of the glycocalyx of DC-SIGN expressing cells on pathogen binding strengthening when interacting with single *Candida* fungal cells. We show that *N*-glycosylation of DC-SIGN plays a previously unidentified role in strengthening DC-SIGN-pathogen binding by providing an additional layer of glycan-mediated lateral interactions between DC-SIGN and other plasma membrane constituents. This most likely results in cross-linking of DC-SIGN to transmembrane glycoproteins that are stably associated with the cortical actin cytoskeleton. This is the first report to our knowledge that documents the direct involvement of the cell membrane glycans in strengthening cell-pathogen binding interactions. Understanding how the plasma membrane nano-environment contributes to the regulation of receptor function will provide novel insights that are relevant for both membrane biologists and immunologists.

## Results

### AFM force spectroscopy to quantify cell-pathogen interaction strength

**Figure 2:**
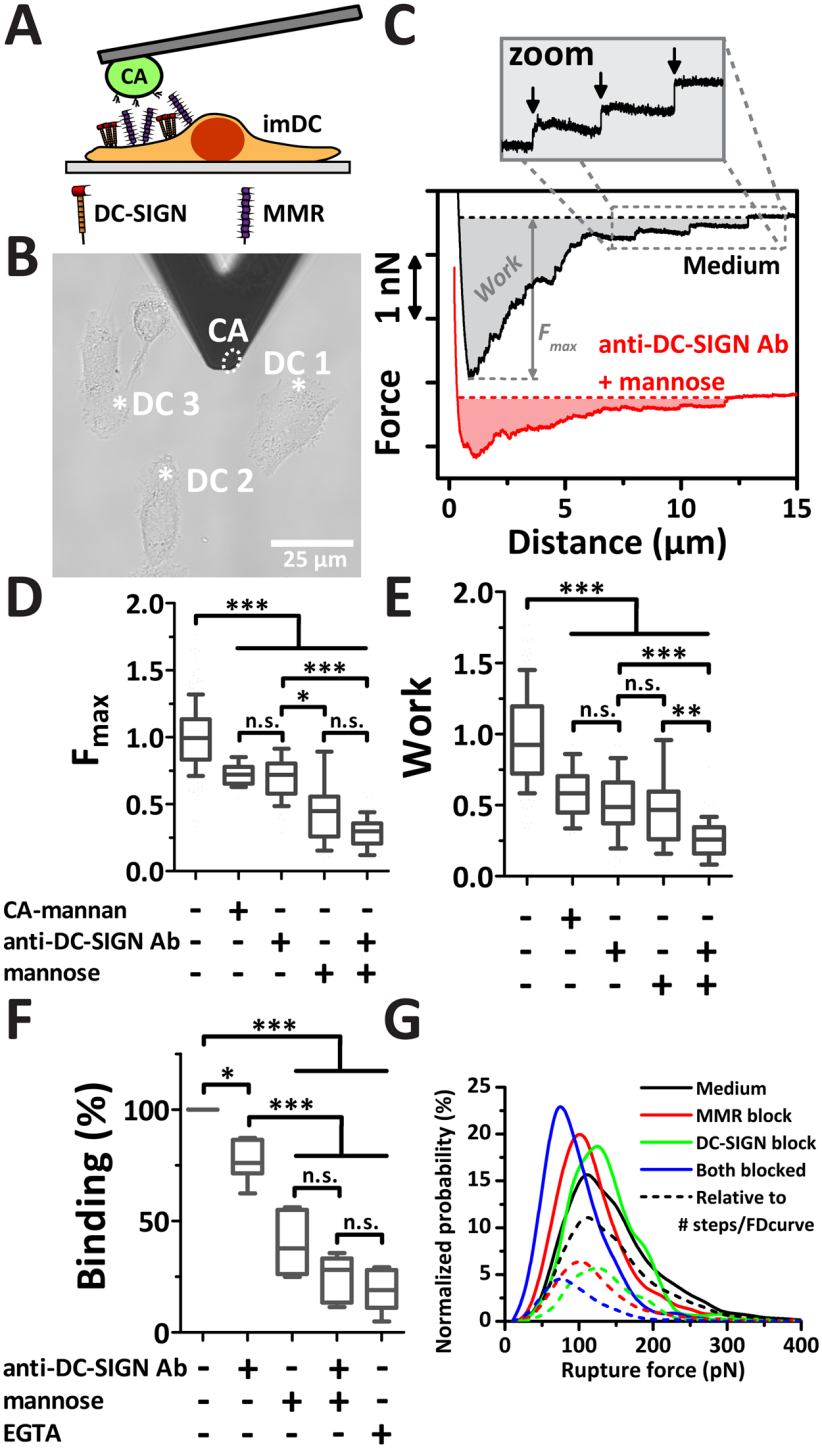
Both DC-SIGN and MMR contribute to the recognition strength of *C. albicans* by immature dendritic cells (imDC). (**A**) Schematic set-up showing a single *C. albicans* cell immobilized on a tip-less AFM cantilever interacting with an imDC expressing different C-type lectins such as DC-SIGN and MMR. (**B**) Example brightfield image that shows how a *C. albicans* cell on the tip (indicating by dashed white ellipse) interacts with different imDCs. The positions at which the *C. albicans* is brought into contact for 10 seconds with three imDCs are indicated by asterisks. (**C**) Example FD-curves of *C. albicans* - imDC interactions are shown of without (medium control) and with specifically blocking DC-SIGN and MMR (with 30 µg/ml anti-DC-SIGN and 30 mM mannose for 30 minutes). In FD-curves the work and F_max_ are measured such as indicated by the shaded area and depth of the curve, respectively. The zoom shows single membrane tether rupture steps (arrows). (**D**,**E**) The relative detachment force (**D**) and work (**E**) needed to detach an *C. albicans* cell from the imDC before and after blocking by CA-mannan, anti-DC-SIGN, mannose, or a combination of mannose and the antibody (N ≥ 3 independent experiments; N ≥ 20 cells; N≥ 20 FD-curves per condition). Significance levels by Mann-Whitney (n.s. = not significant; *p < 0.05; **p < 0.01; ***p < 0.001). (**F**) imDC cells were incubated with FITC-labeled *C. albicans* in the presence or absence of blocking agents; anti-DC-SIGN antibody, mannose and EGTA. The percentage of cells that bound *C. albicans* was calculated by flow cytometry. Data are normalized to medium conditions. (**G**) Loading probability distribution of all rupture steps detected in the FD-curves prior to and after blocking show different distributions that peak at 112 pN (medium), 102 pN (MMR block), 122 pN (DC-SIGN block), and 76 pN (both blocked) (N > 450 rupture steps). The normalized probability plots are shown and the ‘dashed’ curves are scaled relative to the number of rupture steps per FD-curve (arbitrary units).

Our recent AFM studies revealed the interaction affinity between *C. albicans* cells and recombinant single DC-SIGN molecules^34^. To determine the recognition strength of DC-SIGN-mediated pathogen-immune cell interactions at the cell-cell level, we applied AFM-assisted cell-cell force spectroscopy (CCFS)^32, 40^. Therefore, an intact *C. albicans* cell was immobilized underneath the apex of a concanavalin A (ConA)-functionalized tip-less AFM cantilever and subsequently brought into contact with a flat part of a single immature dendritic cell (imDC) attached to a glass coverslip (Fig. 2A). Both MMR and DC-SIGN are known to specifically bind glycan structures in the cell wall of *Candida albicans* ^20, 47^. A combined brightfield-AFM microscope was used to position the *C. albicans* cell over the imDC and subsequent to bring them into contact, all with submicron accuracy (Fig. 2B and Suppl. Movie 1). The interaction strength between imDC and *C. albicans* was measured by quantifying the work and maximum detachment force F_max_ from single force-distance (FD)-curves (Fig. 2C). Many imDC-*Candida* interactions were measured in medium leading to an average F_max_ (Fig. 2D) and work (Fig. 2E). To take donor-dependent heterogeneity of CLR expressions levels into account, we normalized results of different imDCs for which detachment forces varied between ~1–4 nN under unblocked conditions. To test for specificity of the interactions, 150 µg/ml soluble CA-mannan was added 20 min *in situ* to the cells to block DC-SIGN binding^47^ and interactions between *C. albicans* and the same imDCs were probed again. After blocking with soluble CA-mannan, both F_max_ and work are reduced (Fig. 2D,E). To distinguish between the contribution of DC-SIGN and MMR receptors on imDCs that bind *C. albicans*, interactions were specifically blocked with 30 µg/ml anti-DC-SIGN antibody (AZN-D1), 30 mM mannose to block MMR, or a combination of both. To note, these concentrations have been shown to be saturating in previous experiments^20, 47^. In presence of these inhibitors, FD-curves clearly show less work and lower F_max_ (Fig. 2C). Residual interactions after blocking might be due to suboptimal blocking efficiency and some weak a-specific interactions. When specifically looking at the work, we observe that under mechanical load DC-SIGN and MMR equally contribute to binding *C. albicans* (Fig. 2E). Under steady conditions such as measured with flow cytometry, however, MMR seems to be the main contributor to binding (Fig. 2F). In accordance with this, inspection of the detachment force F_max_ also reflects a stronger effect of MMR, which can be explained by a stronger association rate or a higher expression level of the MMR with respect to DC-SIGN.

Discrete rupture steps in the FD-curves (arrows; see Fig. 2C) represent the unbinding of single or multiple receptor-ligand bonds that are disrupted upon detachment of the *C. albicans* from the imDC. Figure 2G shows the analysis of rupture forces before and after blocking DC-SIGN, MMR or both. Rupture forces measured on imDCs in medium conditions show a maximum peak around 110 pN, whereas imDCs blocked for DC-SIGN or MMR show a maximum peak in rupture forces around 130 pN and 105 pN, respectively. Note that when we normalize to the amount of rupture steps per FD curve we see that steps under blocked conditions are much less abundant in FD-curves (Fig. 2G; dotted lines). When both DC-SIGN and MMR are blocked the maximum peak in the rupture forces shifts to 80 pN. These rupture events most likely originate from unspecific binding events with lower affinity. With similar reasoning and having observed that MMR has a stronger effect on the F_max_, we would expect that MMR-*C. albicans* bonds are slightly stronger than DC-SIGN-*C. albicans* bonds; we indeed find rupture forces of 130 versus 105 pN for MMR and DC-SIGN, respectively. Knowing that the detachment force is in the order of 1–4 nN, only tens to hundreds of individual specific bonds are formed between the cell and *C. albicans*.

In summary, by AFM-assisted CCFS we show that both DC-SIGN and MMR are main players in the binding to *C. albicans* with slightly different contributions to the capturing strength under mechanical load. Moreover, these results demonstrate the usefulness of AFM-assisted CCFS to obtain a better insight into the dynamic binding of pathogen and receptor.

### The various domains in the molecular structure of DC-SIGN differently influence the binding strength to *Candida albicans*

**Figure 3:**
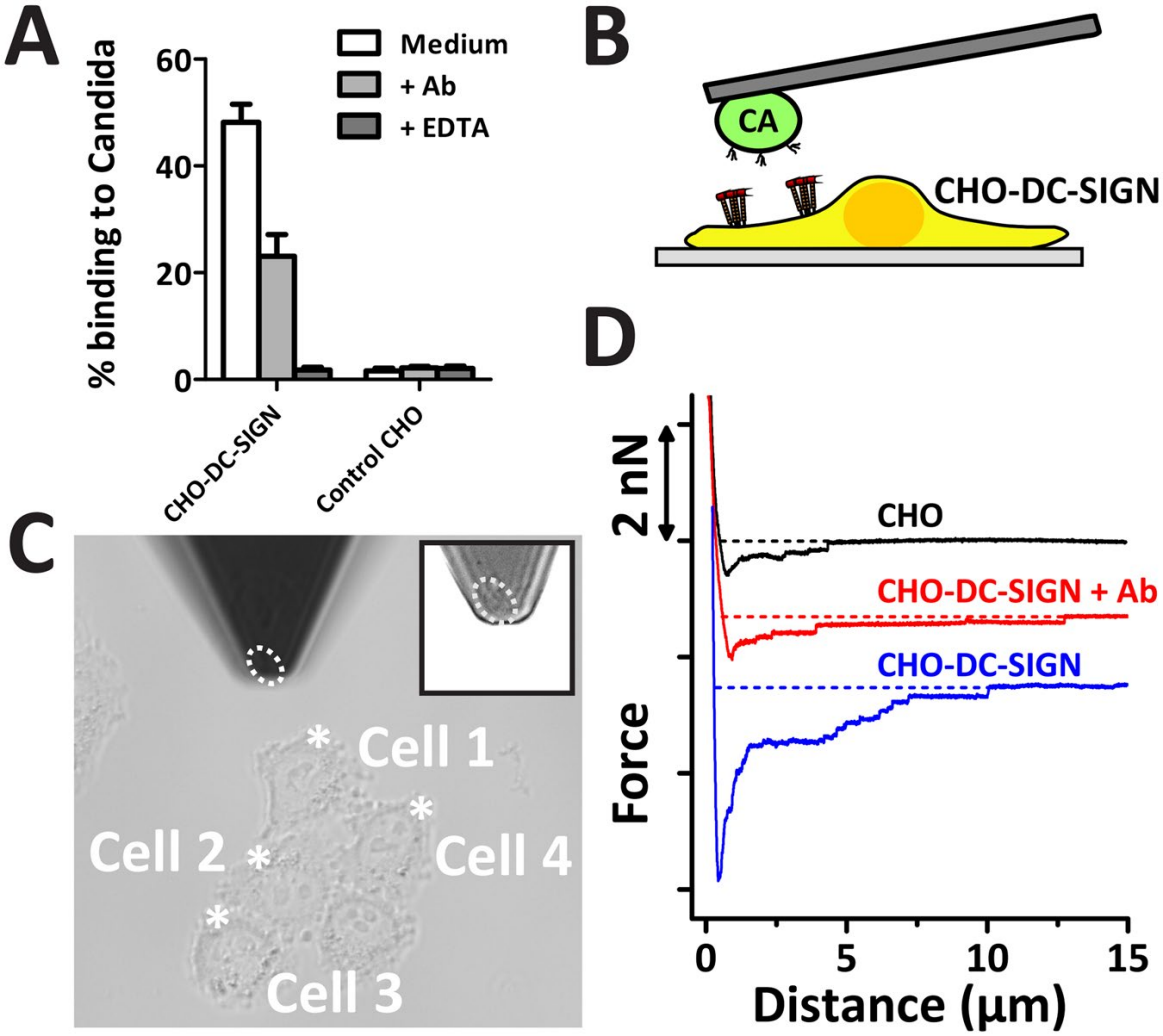
AFM-assisted cell-cell force spectroscopy to measure the interaction strength of single CHO-DC-SIGN cells and *C. albicans*. (**A**) Parental CHO cells and cells expressing DC-SIGN-WT were incubated with FITC-labeled *C. albicans* in the presence or absence of blocking agents; anti-DC-SIGN antibody and EDTA. The percentage of cells that bound *C. albicans* was calculated by flow cytometry. Data are presented as means ± S.D. (**B**) Schematic set-up showing a single *C. albicans* cell immobilized on a tip-less AFM cantilever interacting with a CHO-DC-SIGN cell. (**C**) Example brightfield image that shows how a *C. albicans* cell on the tip (indicating by dashed white ellipse) is positioned to interact with four different CHO-DC-SIGN cells. The positions at which the *C. albicans* is brought into contact are indicated by asterisks. (**D**) Example FD-curves of *C. albicans* interacting with parental CHO, CHO-DC-SIGN-WT with addition of 30 µg/ml anti-DC-SIGN blocking antibody for 30 minutes.

To be able to study the recognition of *C. albicans* by DC-SIGN without interference of MMR and with more expression homogeneity in the cell populations, CHO cells were stably transfected with DC-SIGN wild-type (DC-SIGN-WT) as we previously reported^10, 48^. Ensemble binding studies by flow cytometry of CHO-DC-SIGN-WT cells with *C. albicans* show the specific binding of *C. albicans* by DC-SIGN through blocking by an anti-DC-SIGN antibody or by addition of the Ca^2+^ and Mg^2+^ chelator EDTA (Fig. 3A). To study the recognition of *C. albicans* by DC-SIGN-WT at the single cell level, we used CCFS (Fig. 3B and Suppl. Movie 2), with a single *C. albicans* cell immobilized on the cantilever and brought it into contact for 10 seconds with different CHO cells expressing DC-SIGN-WT (Fig. 3C). Figure 3D shows that after blocking with anti-DC-SIGN, FD-curves have a lower interaction strength (F_max_ and work) and are more similar to FD-curves of parental CHO cells as expected, confirming the applicability of CCFS also in the CHO-DC-SIGN cell system.

**Figure 4:**
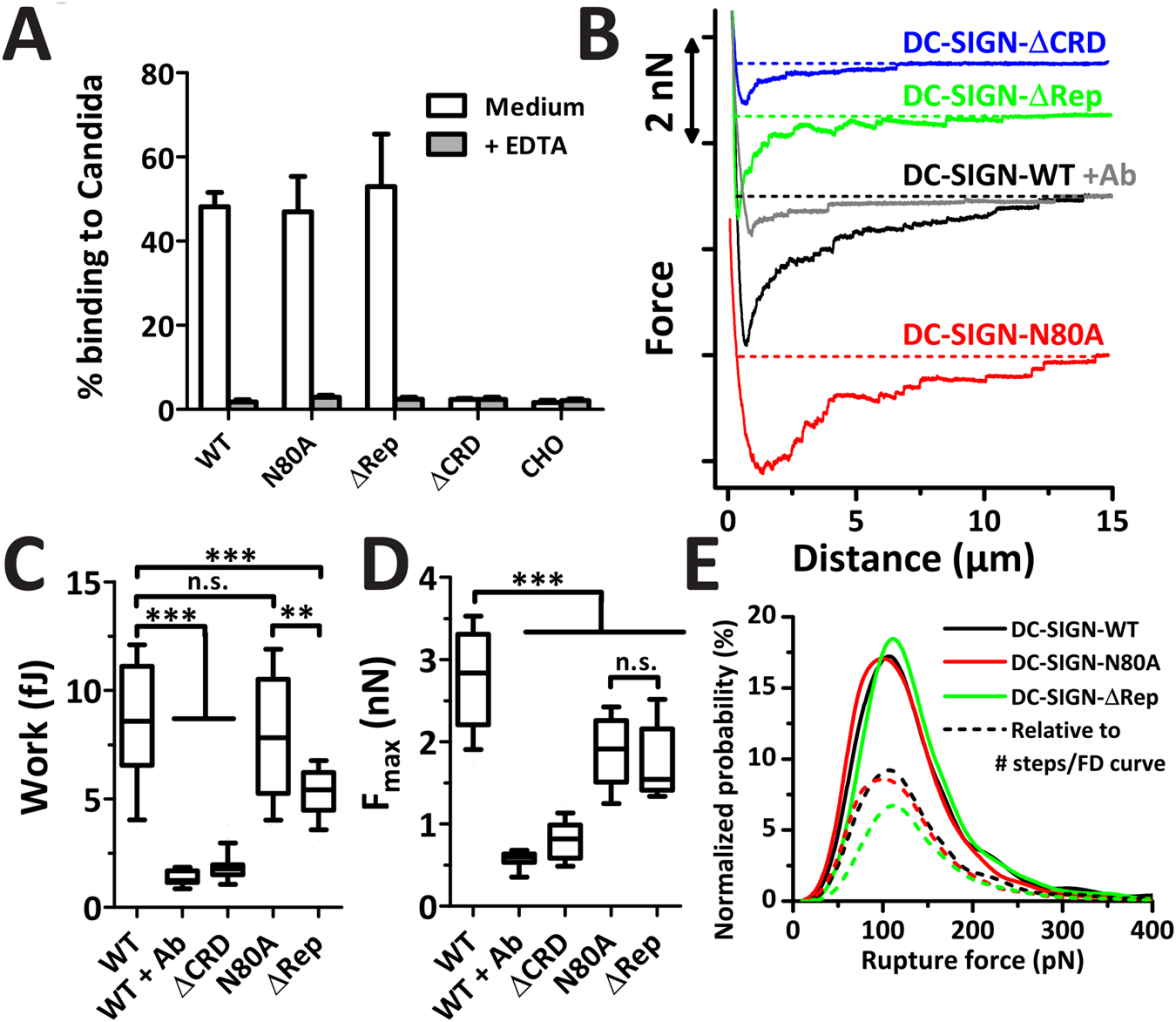
Contribution of the different domains of DC-SIGN to the interaction with *C. albicans*. (**A**) CHO cells expressing the different DC-SIGN mutants were incubated with FITC-labeled *C. albicans* cells in the presence or absence of EDTA. The percentage of cells that bound *C. albicans* was calculated by flow cytometry. Data are presented as means ± S.D. (**B**) Example FD-curves of *C. albicans* interacting with CHO-DC-SIGN-WT with and without blocking Ab, CHO-DC-SIGN-ΔCRD, CHO-DC-SIGN-N80A, or CHO-DC-SIGN-ΔRep cells. (**C**,**D**) The detachment force (**C**) and work (**D**) needed to detach an *C. albicans* cell from the CHO cells stably expressing DC-SIGN-WT before and after blocking by anti-DC-SIGN, and CHO cells stably expressing DC-SIGN-ΔCRD, DC-SIGN-N80A, or DC-SIGN-ΔRep (N ≥ 3 independent experiments; N ≥ 20 cells; N ≥ 20 FD-curves per condition). Significance levels by Mann-Whitney (n.s. = not significant; **p<0.01; ***p<0.001). (**E**) Probability distribution of all rupture steps detected in the FD-curves of DC-SIGN-WT, DC-SIGN-N80A, and DC-SIGN-∆Rep interacting with *C. albicans* show different distributions that all peak around 110 pN (N > 2000 rupture steps). The normalized probability plots are shown and the ‘dashed’ curves are scaled relative to the number of rupture steps per FD-curve (arbitrary units).

Different domains and glycosylation in the molecular structure of DC-SIGN might influence binding to *C. albicans*. To study this, different mutants of DC-SIGN were expressed in CHO cells. Next to DC-SIGN-WT, three mutants were generated: one lacking the CRD domain (DC-SIGN-ΔCRD), one lacking the repeat region (DC-SIGN-ΔRep), and one lacking the single *N*-glycosylation site at amino acid N80, which is mutated to alanine (DC-SIGN-N80A). Cell surface expression of all these constructs was overall the same as confirmed by flow cytometry (Suppl. Fig. S1). Flow cytometry binding studies of these CHO-DC-SIGN cells with *C. albicans* show the specific binding of *C. albicans* on cells expressing DC-SIGN with an intact CRD domain only (Fig. 4A). Similar to these flow cytometry studies, CCFS studies show that CHO cells expressing DC-SIGN with a CRD domain, *i.e*., DC-SIGN-WT, -N80A, and -ΔRep, interact stronger with *C. albicans* cells (Fig. 4B–D) than CHO-DC-SIGN-WT cells blocked with anti-DC-SIGN or CHO-DC-SIGN-ΔCRD, the latter two showing both only background level of binding (Fig. 4B). Next,we plotted work as well as F_max_ to compare DC-SIGN-WT with the mutants (Fig. 4C,D). As expected, both Ab-blocked DC-SIGN-WT and DC-SIGN-ΔCRD showed negligible work and F_max_ values. Interestingly, while DC-SIGN-WT and DC-SIGN-N80A exhibited the same work (Fig. 4C), the average F_max_ value was lower for the N-glycosylation-impaired mutant (Fig. 4D). This could be explained by considering that although a similar amount of DC-SIGN molecules bound to *C. albicans*, they are less capable of withstanding the pulling forces exerted by the AFM cantilever. This could explain why the F_max_ is lower in the case of DC-SIGN-N80A.

When we compare the results of cells expressing DC-SIGN-ΔRep with those expressing DC-SIGN-WT or -N80A, CHO-DC-SIGN-ΔRep cells are less capable binding *C. albicans*, as reflected in a lower work and detachment force compared to DC-SIGN-WT (Fig. 4C,D). However, when comparing to DC-SIGN-N80A, F_max_ is similar (Fig. 4D), but the work is significantly less for ΔRep (Fig. 4C). Earlier studies showed that DC-SIGN without its repeat domain cannot tetramerize^24, 49^. Tetramerization of DC-SIGN is known to contribute to a higher binding capacity for different ligands^49^, which could explain the lower work. Our measurements allow to analyze the strength of single interactions in FD-curves (Fig. 4E); maximum peaks are found around 110 pN rupture force in all cases, with no clear differences. The explanation for this observation is that the rupture forces measured here are a mix of ruptures depending on the affinity of bonds and of membrane tether ruptures that depend on retraction speed and membrane properties, which are the same for the different CHO-DC-SIGN cell lines^33, 50^. The lower abundance of rupture steps per FD-curve for CHO-DC-SIGN-ΔRep in contrast to WT and N80A relates to its lower adhesion as reflected in the work (Fig. 4B,C). In summary, the various domains of DC-SIGN seem to differently affect the overall adhesion to *C. albicans*; the CRD domain crucial for binding whereas the repeat region and *N*-glycosylation site clearly contribute to the mechanic strength.

### Tetramers of DC-SIGN are stronger anchors of membrane tethers than monomers

**Figure 5:**
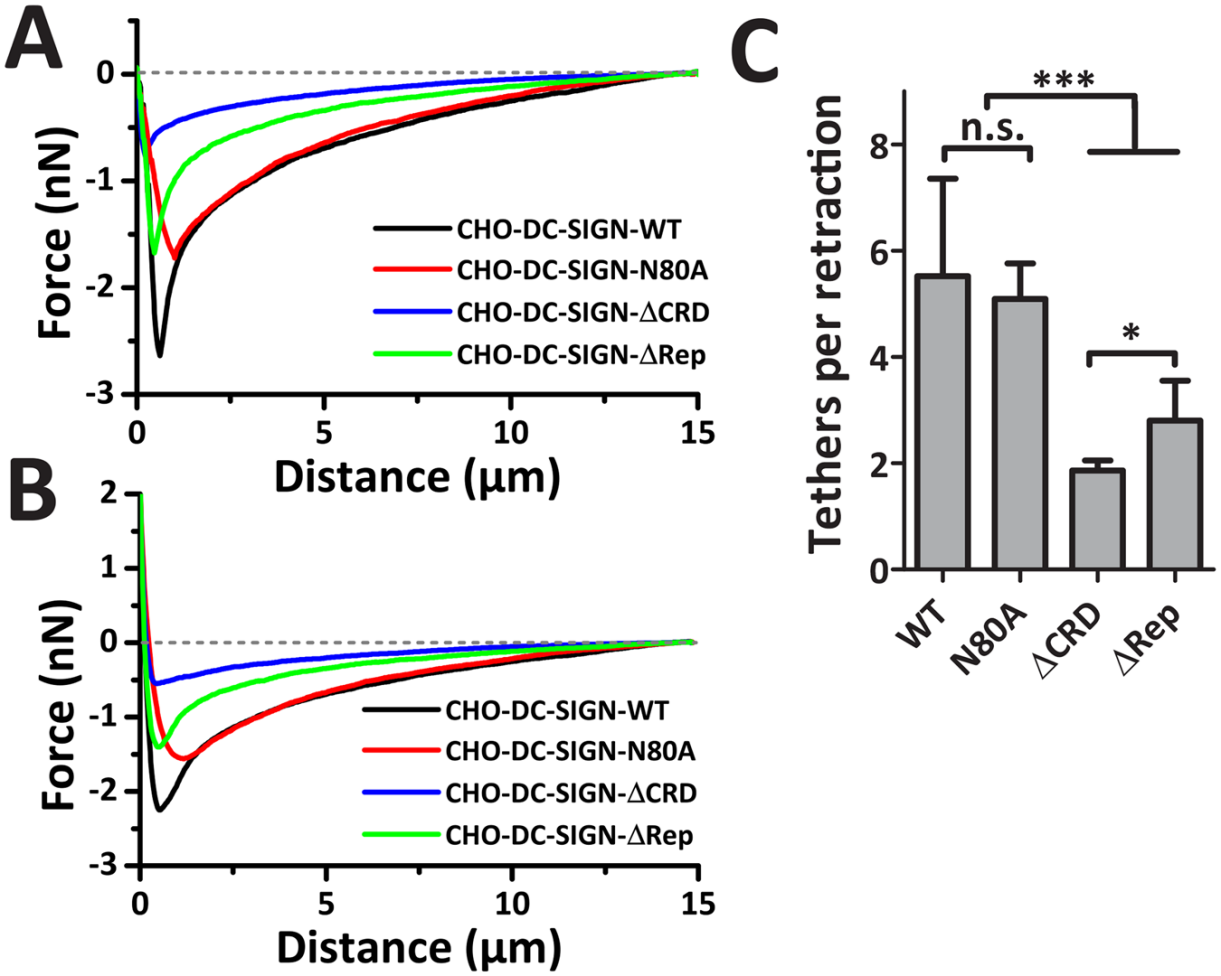
Tetrameric DC-SIGN contributes to more membrane tethers per retraction. (**A**,**B**) Averaged FD-curves of CHO cells expressing DC-SIGN-WT, DC-SIGN-N80A, DC-SIGN-ΔCRD, and DC-SIGN-ΔRep (N ≥ 20 cells) were generated by averaging N ≥ 100 FD-curves. For averaging, FD-curves were aligned on the maximum detachment peak (**A**) or on the 2 nN position (**B**). (**C**) The number of membrane tether rupture events observed per retraction of a *C. albicans* cell being detached from a CHO cell expressing either DC-SIGN-WT, DC-SIGN-N80A, DC-SIGN-ΔCRD, or DC-SIGN-ΔRep (N ≥ 20 cells; N ≥ 100 FD-curves). Data are presented as means ± S.D.

To better determine and quantify whether DC-SIGN-WT and its mutants differ in their ability of withstanding pulling forces, FD-curves for each cell line were averaged and aligned at the x-axis position of F_max_ (Fig. 5A) or at the position of maximum contact force of 2 nN (Fig. 5B). In Fig. 5B the presence of a sharper dip in the FD-curve for DC-SIGN-WT, which is less pronounced for DC-SIGN-N80A, is clearly visible. To note is that averaging at the F_max_-position (Fig. 5A) keeps the average detachment force and work corresponding to the data of Fig. 4C and D, whereas averaging at the 2nN-position (Fig. 5B) ‘smears’ the sharp peak^33^. The averaged FD-curves of DC-SIGN-ΔCRD show the signature of non-specific background adhesion, which is small. Inspecting the depth of the minimum peak shows that interactions of DC-SIGN-ΔRep and DC-SIGN-N80A have similar depths, which are both lower than that of DC-SIGN-WT (Fig. 5A,B) and relates to the differences in F_max_ found (Fig. 4D). However, in the case of DC-SIGN-N80A and DC-SIGN-WT more interactions are present over longer retraction distances, 2–15 µm away from the cell (Fig. 5A,B). This implies that for these cells a larger part of the cellular membrane is pulled along with the *C. albicans* upon retraction of the AFM cantilever. These pieces of membrane that are pulled from the CHO-DC-SIGN-WT and -N80 cell are probably anchored to the *C. albicans* by DC-SIGN molecules. The disconnection of these anchors upon pulling results in small rupture steps in the FD-curves (see Fig. 2C). We previously demonstrated that at distances >5 µm most of these events result from disconnecting membrane tethers^33^. Therefore, we next analyzed the mean number of membrane tethers in these FD-curves and found that for DC-SIGN-ΔRep membrane tethering is comparable to non-specific (background) membrane tethering of DC-SIGN-ΔCRD cells, which are not capable of forming DC-SIGN-anchored membrane tethers because of the absence of the CRD-domain (Fig. 5C). Knowing that the lifetime of a membrane tether relates to the lifetime of the bond(s) anchoring it, we conclude that less membrane tethers relate to weaker bonds^50^. Therefore, although DC-SIGN-ΔRep is expressed at similar levels as DC-SIGN-WT, these cells have a lower intrinsic adhesion strength F_max_ and are less capable of forming DC-SIGN-anchored membrane tethers, which can be explained by DC-SIGN-ΔRep being monomeric whereas DC-SIGN-WT and -N80A forming tetramers. These results collectively show that DC-SIGN-WT and -N80A have the same capacity to form membrane tethers, which is higher than the ΔRep mutant. This also indicates that tetramerization but not *N*-glycosylation has a role in membrane tethering formation.

### Glycosylation of DC-SIGN contributes to a stronger connection to the actin cortex

**Figure 6:**
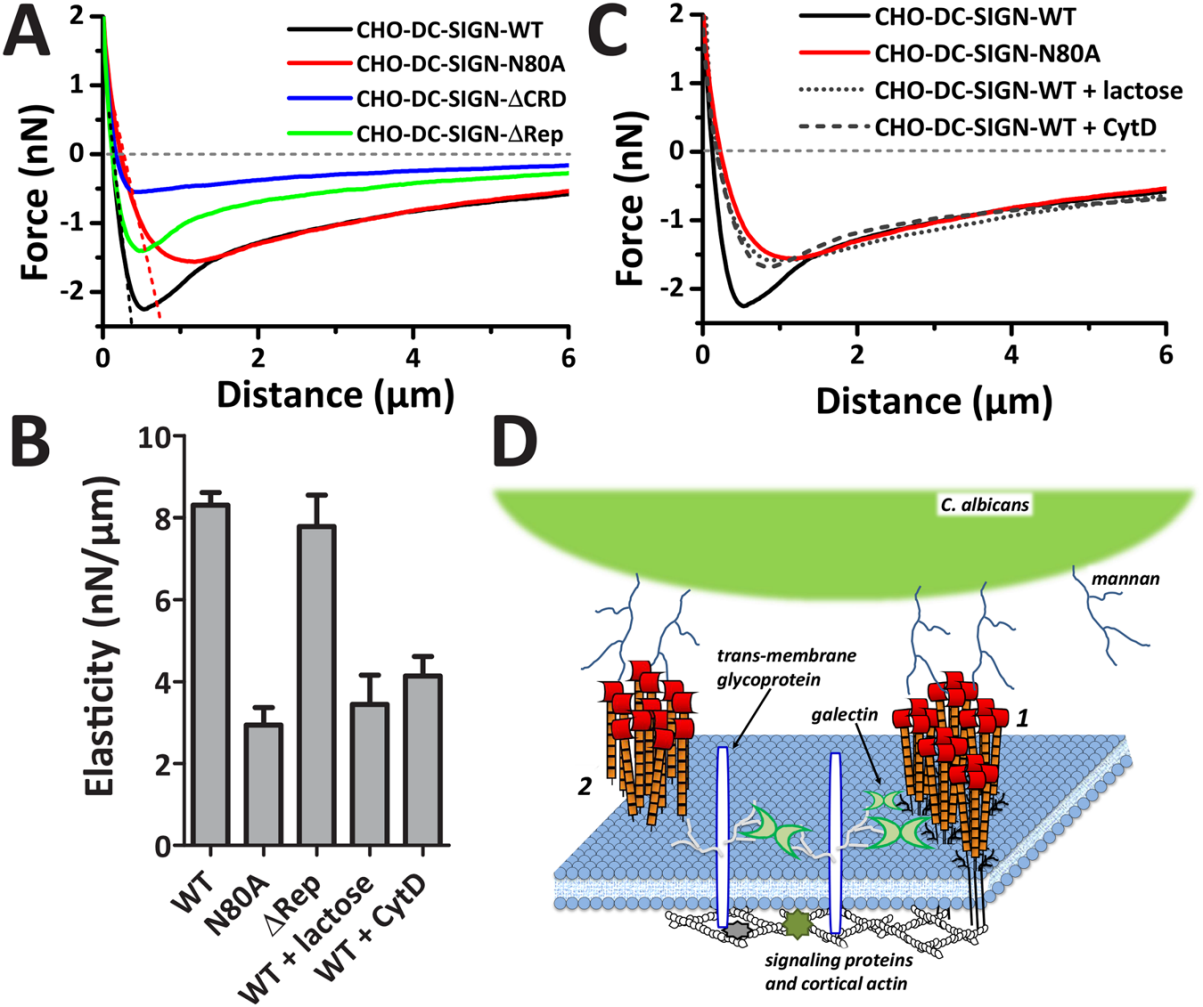
*N*-glycosylation of DC-SIGN contributes to a more resistant adhesion site. (**A**) Zoom in to the averaged FD-curves of CHO cells expressing DC-SIGN-WT, DC-SIGN-N80A, DC-SIGN-ΔCRD, and DC-SIGN-ΔRep of Fig. 5B. Dotted lines indicate semi-linear fits to the slope before of the retraction curve until maximum force is reached at F_max_ (*i.e*., contact region) from averaged retraction FD-curves DC-SIGN-WT and DC-SIGN-N80A. (**B**) The elasticity for cells expressing the different DC-SIGN-constructs, CHO-DC-SIGN-WT treated by lactose or CytD; measured by fitting the linear slope presented in (**A**). (**C**) Averaged FD-curves of CHO-DC-SIGN-WT cells untreated, cultured for 24 h in 100 mM lactose, or treated with 10 µM of the actin polymerization inhibitor CytD compared to CHO-DC-SIGN-N80A cells (N ≥ 15 cells; N ≥ 80 FD-curves). (**D**) Cartoon depicting the interaction between *C. albicans* and DC-SIGN and the role played by the *N*-glycans in strengthening pathogen-binding. When DC-SIGN nanoclusters bear *N*-glycans (1), they can laterally interact with transmembrane proteoglycans such as CD44, through galectin-mediated cross-linking. Molecules such as CD44 are almost stably associated with cortical actin, thus providing a structural scaffold that might facilitate DC-SIGN-actin interactions. The absence of the *N*-glycans (such as in N80A or after lactose treatment) prevents DC-SIGN-galectin interaction (2), thus hampering the possibility to firmly link to the actin cytoskeleton. The *N*-glycan-galectin network at the outer leaflet of the plasma membrane and, therefore, adds an extra level of binding strengthening during DC-SIGN-pathogen interactions, which is distinct yet connected to the cortical actin polymerization.

We demonstrated so far that *N*-glycosylation of DC-SIGN is important for the receptor to withstand pulling forces, but has no role in membrane tethering during DC-SIGN-*Candida* detachment. Therefore, we sought to investigate whether *N*-glycosylations plays a role in the overall cell stiffening in response to pathogen binding and mechanical retraction. Analysis of averaged FD-curves can provide insight in the elasticity of the microenvironment of the receptor binding sites^33, 51^. Therefore, we fitted the (semi-)linear regime of averaged FD-curves over a distance from 0 µm to the distance at which the highest force acts on the bonds; *i.e*. the distance at which we measure F_max_ (Fig. 6A; dotted lines), thus obtaining elasticity values. This gives insight in how DC-SIGN-expressing cells cope with forces that build up at the adhesion site with *C. albicans*. We found that the elasticity of DC-SIGN-WT bonds holding to *C. albicans* is almost 3-fold higher than that of DC-SIGN-N80A (Fig. 6B), whereas DC-SIGN-ΔRep has a similar elasticity as DC-SIGN-WT (Fig. 6B). Interestingly, since the mutant DC-SIGN-ΔRep maintained its *N*-glycosylation, the elasticity measurements suggest that glycan interactions may indeed play a role in strengthening cell-cell contacts under mechanical load.

We showed before that pretreatment of CHO-DC-SIGN-WT cells with 100 mM lactose overnight can effectively dissolve the lateral interactions of galectins with glycans present on the cellular membrane, resulting for example in changes of nanoscale lateral mobility behavior of DC-SIGN^29^. Here, we treated CHO-DC-SIGN-WT cells with lactose and analyzed the adhesion to *C. albicans* by AFM-assisted CCFS. We observe that FD-curves of lactose-treated CHO-DC-SIGN-WT cells have a similar unbinding ‘signature’ as CHO-DC-SIGN-N80A (Fig. 6C). Moreover, they also exhibited a lower elasticity (Fig. 6B) and the absence of a sharp F_max_ peak (Fig. 6C). Interestingly, exactly the same alteration in unbinding signature (Fig. 6C) and reduced cell elasticity (Fig. 6B) were observed when CHO-DC-SIGN-WT cells were pre-treated with cytochalasin D (CytD) to disturb actin polymerization. This indicates a potential role of galectins in stabilizing DC-SIGN contacts probably due to indirectly linking them to the actin cortex.

## Discussion

In this study, exploiting AFM-assisted adhesion measurements between two individual cells, we measured the strength with which a single cell expressing the pathogen-recognition receptor DC-SIGN binds a single fungal *Candida albicans* cell and we determine a novel role for DC-SIGN *N*-glycosylation. We find that during dynamic interactions DC-SIGN accounts for 50% of the initial binding of *Candida* to imDCs. Moreover, the organization of DC-SIGN on the cellular membrane by tetramerization as well as glycan-mediated lateral interactions is important to strengthen adhesion to pathogenic fungal cells under mechanical load. This study, therefore, puts forward a very interesting role for the glycocalyx as extracellular cytoskeleton contributing, possibly in connection with the intracellular actin cytoskeleton, to optimize strengthening of cell-pathogen interactions in the presence of mechanical forces.

Here, we wanted to gain more insight in the dynamical interaction strength of DC-SIGN recognizing *C. albicans*. In previous experiments using flow cytometry, we have demonstrated that imDCs bind and internalize *C. albicans* through the CLRs DC-SIGN and MMR^20, 47^. These earlier experiments showed that MMR might be the main contributor to binding, where DC-SIGN plays a secondary role. By contrast, our CCFS results now show that DC-SIGN contributes up to 50% to preserve binding to *Candidas* under dynamic conditions. Except of a difference in scale, *i.e*. 100,000 s vs. single cells, a more prominent difference is that flow cytometry is performed under static conditions whereas CCFS measures adhesion under mechanical load comparable to the physiological conditions during infection of the host. Thus, the role of DC-SIGN on imDCs in capturing *Candidas* might become more apparent when mechanical forces act at the interaction site, which is at the order of only tens of µm² and depends on dwelling time and *Candida* size. By applying physiological pulling forces on the pathogens using an AFM cantilever, we determined a capturing strength of 1–4 nN by DC-SIGN molecules after only 10 seconds of contact with single unbinding events of 105–110 pN each, probably related to the high discriminative binding capacity of DC-SIGN for pathogenic carbohydrates^22, 24, 34^. These interactions forces are comparable though slightly lower than those of *C. albicans* and macrophages that range between 0.25–3.0 nN for the same interaction time and can primarily be attributed to MMR-mediated interactions^43^. However, MMR expression level differences might differ between these macrophages and our imDCs making a fair comparison difficult.

Earlier studies attributed the binding of *C. albicans* to the carbohydrate recognition domain (CRD) of DC-SIGN and to the α-(1,2) branched mannose residues^21, 47^. Moreover, we recently showed by AFM force spectroscopy studies that slight differences in the *N*-mannan structure of *Candida*, such as, the absence or presence of a phosphomannan side chain, can be recognized by DC-SIGN^34^. Next to the specific affinity of DC-SIGN’s CRD domain for specific pathogenic sugars, another level of binding strength control exploited by DC-SIGN is its intrinsic tetramerization^22^ and its tendency to form nanoclusters on the cellular membrane^9^. Structural studies showed that DC-SIGN forms tetramers through its repeat regions bringing four CRDs in proximity^49, 52^. In the study described here, it is likely that all CRDs bind ligands present in the mannan structures of *C. albicans* outer cell wall, and thereby support cooperative affinity of the bonds, *i.e*., avidity. Indeed, we observed that mutant DC-SIGN molecules expressed on CHO cells without their repeat region (DC-SIGN-∆Rep) adhered weaker to *C. albicans* (Fig. 4). Earlier studies showed that DC-SIGN-∆Rep is not in nanoclusters and is present as monomers on the cellular membrane^10, 23^. Therefore, it may lack the capacity to increase binding strength by cooperative binding and thus shows a weaker adhesion. Moreover, when present on the plasma membrane as tetramer, *i.e*., as WT or N80A, DC-SIGN is more prone to form membrane tethers over long distances (Fig. 5), probably, because a tetramer of DC-SIGN anchoring a membrane tether with four bonds has a longer lifetime than a monomeric DC-SIGN bond^53^. All these subtle differences between DC-SIGN mutants could not be picked up by studying binding by flow cytometry, *i.e*., without any mechanical force involved, highlighting the power of AFM-assisted CCFS.

Recent work of our group provided evidence that *N*-glycan-mediated membrane micropatterning plays a role in organizing DC-SIGN molecules in clathrin-enriched regions to induce phagocytosis of pathogens^29^. Removal of *N*-glycans from DC-SIGN or neutralization of DC-SIGN-galectin interactions by lactose indicated that galectin-9 might be the glycan-binding protein that links DC-SIGN to the trans-membrane glycoprotein CD44^29^. Both galectin-9 and CD44 were found together with DC-SIGN on imDCs phagosomes^54^. In the study described here, we explored the correspondence of *N*-glycan-mutated DC-SIGN (DC-SIGN-N80A) binding to *C. albicans* by dynamically pulling the fungal cell away from the DC-SIGN-expressing cell (Figs 5 and 6). When the *N*-glycosylation site is abrogated or binding by galactin-9 blocked by lactose, we observe that the adhesion structure is less stable: *i*) the detachment force F_max_ is lower, and *ii*) the elasticity is smaller when we pull on the adhesive structure formed between cell and pathogen, probably because DC-SIGN loses its indirect linking to the actin cortex (Fig. 6D). Earlier, we hypothesized that galectin-9 connects DC-SIGN to CD44 through glycan-protein-mediated linkages and may thereby indirectly link DC-SIGN to the actin cytoskeleton^29^, because CD44 is known to associate with actin^13^. This may explain how DC-SIGN can be maintained in clathrin-rich regions and support phagocytosis of pathogens. The results showed here support this hypothesis.

In summary, we demonstrate to the best of our knowledge for the first time how the strength of AFM can be exploited to bring a single *C. albicans* cell into contact with a single primary phagocytic cell of the immune system to study the recognition of this pathogen by the pathogen-recognition receptor DC-SIGN expressed on an imDC. We show that DC-SIGN exploits different mechanisms to strengthen adhesion after recognition. Apart from tetramerization and the formation of microdomains of the receptor that supports a longer lifetime of bonds, also *N*-glycan-mediated linkages help to support firm binding of the pathogen probably by indirect linkage to the actin cortex through membrane-organizing galectins that bind *N*-glycosylated DC-SIGN at one side and CD44 or other cell adhesion molecules on the other side. This dynamic strengthening of the adhesion by the model proposed here might facilitate internalization of the pathogen upon binding. Moreover, our single cell-cell experiments show that the immune receptor DC-SIGN is very well capable of recognizing pathogens under dynamic conditions and thus protecting the human body from fungal infections. Finally, the interesting observation that *N*-glycosylation of a CLR might support pathogen recognition could include *N*-glycan as future emerging targets in anti-microbial therapies, while also conveying novel concepts to the cell adhesion and mechano-biological fields.

## Methods

### Antibodies and blocking agents

Anti-DC-SIGN (AZN-D1) mouse monoclonal antibody was produced as already described^15^. Anti-DC-SIGN antibody DCN46 was from BD Pharmingen. Mannan derived from *Candida albicans* (CA-mannan) and *Saccharomyces cerevisiae* (SC-mannan) were kind gifts from Dr. G. Kogan (Institute of Chemistry of Slovak Academy of Sciences, Bratislava, Slovakia)^55^. The Ca^2+^/Mg^2+^-chelators EDTA and EGTA as well as the lactose and were from Sigma Aldrich.

### Candida albicans

*C. albicans*, strain CAI-4 serotype A, a well described clinical isolate^56^, were grown as reported elsewhere^57^. Briefly, starter cultures were grown in 10 ml of Sabouraud broth overnight at 30 °C. 1 ml of overnight culture was inoculated into 100 ml of Sabouraud broth and cultured at 30 °C until log phase is reached. After two washes with pyrogen-free saline by centrifugation at 1500 × g, the number of yeast cells was counted in a hemocytometer and resuspended at 1 × 10^8^ cells/ml. Heat-inactivation was at 56 °C for 1 h. Yeast cell suspensions were kept frozen at −80 °C until used. Labeling of *Candida* cells was performed as follows: yeast cells were resuspended to 2 × 10^8^ cells/ml, in 0.01 mg/ml FITC (Fluka) in 0.05 M carbonate-bicarbonate buffer (pH 9.5). After incubation for 15 min at room temperature in the dark, FITC-labeled *Candida* cells were washed twice in PBS containing 1% BSA^20^.

### Cell culture

CHO cell lines stably expressing DC-SIGN wild-type, DC-SIGN-N80A, -ΔRep, and -ΔCRD mutants, were established by Lipofectamin 2000 (Invitrogen) transfection, and were cultured in Ham’s F-12 medium (LabClinics) supplemented with 10% heat-inactivated FBS (Invitrogen), 1% Antibiotic Antimycotic Solution (GE Healthcare Life Sciences), and 0.5 mg/ml of the aminoglycoside antibiotic G418 (Invitrogen)^58^. To note, the use of CHO cells expressing DC-SIGN wild-type or mutant has been already validated in previous publications^9, 10, 20, 29, 47, 48^. Human immature dendritic cells (imDCs) were generated from peripheral blood monocytes of healthy donors as reported elsewhere^15^, and cultured in RPMI, 10% FBS, 1% antibiotic-antimycotic solution supplemented with 800 U/ml granulocyte-macrophage colony-stimulating factor (GM-CSF), and 500 U/ml Interleukin-4 (IL-4).

### Candida binding studies

The binding of stable CHO transfectants expressing DC-SIGN to *Candida* yeast cells was measured by flow cytometry using the FACS Calibur (BD Biosciences) and performed as earlier described^15, 20^. To test the effect of various reagents on ligand binding the following reagents were used: different carbohydrates at 150 μg/ml, EDTA at 2 mM, anti-DC-SIGN antibody AZN-D1 at 30 μg/ml. Incubations were performed in TSM (20 mM Tris, 150 mM NaCl, 1 mM CaCl_2_, 2 mM MgCl_2_, pH 8.0), and 1% bovine serum albumin, as already published^20^. FITC-labeled *Candida* was added in a cell/yeast ratio of 1:10. After 20 min of incubation at 37 °C, cell-yeast conjugates were analyzed by flow cytometry.

### AFM and cantilevers

Force measurements were made in force-distance mode using a combined BioScope Catalyst AFM (Bruker, Santa Barbara, CA) and an inverted 3-channel Leica TCS SP5 II confocal laser-scanning microscope equipped with an 40 × 0.85NA air objective and Hamamatsu (ORCA-05G) brightfield camera. The AFM was equipped with a temperature controller to keep temperature stable at 37 °C. Triangular tipless gold-coated silicon-nitride cantilevers were used with nominal spring constants of 0.06 N/m as given by the manufacturer (NP-O type D, Bruker). Each cantilever was calibrated before use by the thermal noise calibration method^59, 60^.

### Immobilization of C. albicans on AFM cantilevers

*C. albicans* cells with a diameter of 4.7 ± 0.4 µm were attached to tipless AFM cantilevers that were coated by concanavalin A (ConA; Sigma) essentially as described earlier^33^. ConA-coated cantilevers were prepared as follows: cantilevers were cleaned by immersion in pure 18 M sulphuric acid (Sigma) for 1 hour, then thoroughly rinsed with Milli-Q water, ethanol and after a final rinse in Milli-Q water let to dry. Following an overnight incubation at 4 °C in ConA at 2 mg/ml in PBS, the cantilevers were rinsed and stored in PBS for no more than one day. Intact *C. albicans* cells kept in TSM were seeded into the liquid chamber of the AFM containing serum-free culture medium plus 10 mM HEPES. The optical microscope mounted on the AFM was hereby used to position the ConA-functionalized cantilever over a target *C. albicans* cell. Subsequently, contact was established between cantilever and cell for at least 10 seconds. During this time, the applied indentation force was kept constant at about 5 nN. Upon retraction, the successful pick-up was readily scored by visual inspection, and, in these events, the fungal cell was positioned on the apex of the cantilever (Fig. 2B).

### Adhesion measurements and analysis

The cantilever with the *Candida* cell was brought into contact with imDCs or CHO cells expressing DC-SIGN for a preset period of time (an interaction time of 10 seconds) on the Catalyst AFM. During this time, a force was exerted on the cell of 2 nN. Upon retraction at 15 µm/s, the forces acting on the cantilever were recorded as a function of displacement of the cantilever from the substrate in force-distance (F-D) curves. DC-SIGN-*Candida* rupture forces were determined directly from rupture steps in the FD-curves (see Fig. 2C). The detachment force (F_max_) was measured by determining the force difference between minimum force and the baseline when zero force acts on the cantilever. The area enclosed by the zero-force baseline and the FD-curve (Fig. 2C) was taken as a measure for the work (*W* = *F*·*d*) needed to detach the contact^61^. Specificity was verified by an *in situ* incubation with the DC-SIGN-specific monoclonal antibody AZN-D1 (30 µg/ml), soluble CA-mannan (150 µg/ml), and EDTA (2 mM) for 30 minutes. *Candida* cells were brought into contact with flat parts at the periphery of imDCs or CHO cells. Whenever appropriate, cells were treated with growth medium containing 100 mM of lactose for a minimum of 24 h, to disrupt glycan-dependent interactions mediated by cell surface galectins^29, 62^. To disrupt the actin cytoskeleton, cells were treated with 10 µM cytochalasin D for 30 min.

Acquired data were exported from the BioScope Catalyst by the NanoScope Version 8.15 software and further analyzed in MATLAB and IgorPro using in-house analysis macros^33, 37^. Data were analyzed in a similar way as described earlier^33^. In short, rupture steps were detected by the macro, steps that were at distances >5 µm from the cell were defined as membrane tethers, FD-curves were averaged at the position of maximal adhesion (F_max_) or at 2 nN force (contact force), and stiffness was calculated by a linear fit to the 1nN-to-F_max_ regime. Origin and GraphPad Prism were used for fitting and statistics.

### Data availability

The datasets generated during and/or analyzed during the current study are available from the corresponding author on reasonable request.

## Electronic supplementary material

Supplementary material

Supplementary Movie 1

Supplementary Movie 2
