# Supplementary material for: N-glycan mediated adhesion strengthening during pathogen-receptor binding revealed by cell-cell force spectroscopy
Source: Sci Rep. 2017 Jul 27;7:6713. doi: 10.1038/s41598-017-07220-w (PMC5532264; doi:10.1038/s41598-017-07220-w)
Supplement: Supplementary file 1 — Supplementary material [file 41598_2017_7220_MOESM1_ESM.pdf]

Supplementary information:

N-glycan mediated adhesion strengthening during pathogen-receptor binding revealed by cell-cell force spectroscopy

Joost te Riet, Ben Joosten, Inge Reinieren-Beeren, Carl G. Figdor and Alessandra Cambi

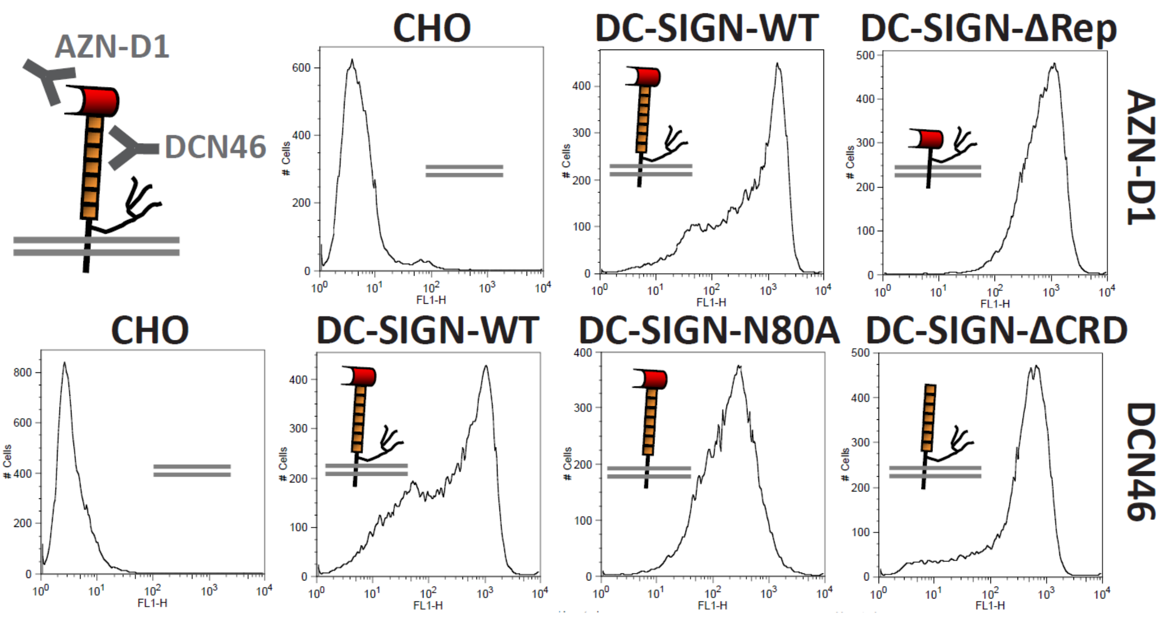

Supplementary Figure 1: The expression of DC-SIGN on different CHO cell lines quantified by flow cytometry. The expression of different DC-SIGN constructs in stably expressed CHO-cells measured by flow cytometry. DC-SIGN was labeled by the anti-DC-SIGN antibodies AZN-D1 (mIgG1) directed against the CRD domain and DCN46 (mIgG2b) against the repeat region. The mean fluorescence intensity is indicated. Parental CHO cells function as negative control. The binding domain of the antibodies and the presence of the different domains are schematically indicated, the two different types of anti-DC-SIGN antibodies used specifically recognize the CRD domain (AZN-D1) and repeat region (DCN46).

Movie captions:

Supplementary Movie 1: AFM-assisted CCFS measurement of a *Candida albicans* cell and an imDC. A *Candida albicans* cell attached underneath an AFM cantilever is brought twice into contact with a flat part of an imDC for 5 sec. In the medium some debris is visible. A scale bar and time stamp are provided in the movie.

Supplementary Movie 2: AFM-assisted CCFS measurement of a *Candida albicans* cell and a CHO-DC-SIGN-WT cell. A *Candida albicans* cell attached underneath an AFM cantilever is brought 3 times into contact with a flat part of a CHO-DC-SIGN-WT cell for 5 sec. A scale bar and time stamp are provided in the movie.
